# Altered thermal preferences of infected or immune-challenged *Aedes aegypti* and *Aedes japonicus* mosquitoes

**DOI:** 10.1038/s41598-024-63625-4

**Published:** 2024-06-05

**Authors:** David O. H. Hug, Raphaela Gretener-Ziegler, Raffael I. Stegmayer, Alexander Mathis, Niels O. Verhulst

**Affiliations:** National Centre for Vector Entomology, Institute of Parasitology, Vetsuisse and Medical Faculty, University of Zürich, Winterthurerstr. 266A, 8057 Zurich, Switzerland

**Keywords:** Behavioural ecology, Infectious diseases, Infectious diseases

## Abstract

Temperature is a critical factor shaping physiology, life cycle, and behaviour of ectothermic vector insects, as well as the development and multiplication of pathogens within them. However, the influence of pathogen infections on thermal preferences (behavioural thermoregulation) is not well-understood. The present study examined the thermal preferences of mosquitoes (*Aedes aegypti* and *Ae. japonicus*) infected with either Sindbis virus (SINV) or *Dirofilaria immitis* over 12 days post exposure (p.e.) or injected with a non-pathogenic Sephadex bead over 24 h in a thermal gradient (15–30 °C). SINV-infected *Ae. aegypti* preferred 5 °C warmer temperatures than non-infected ones at day 6 p.e., probably the time of highest innate immune response. In contrast, *D. immitis*-infected *Ae. japonicus* preferred 4 °C cooler temperatures than non-infected ones at day 9 p.e., presumably a stress response during the migration of third instar larvae from their development site to the proboscis. Sephadex bead injection also induced a cold preference in the mosquitoes but to a level that did not differ from control-injections. The cold preference thus might be a strategy to escape the risk of desiccation caused by the wound created by piercing the thorax. Further research is needed to uncover the genetic and physiological mechanisms underlying these behaviours.

## Introduction

Small ectodermic insects like mosquitoes are highly dependent on environmental factors. Temperature for example influences their physiology, life cycle and behaviour^1^. Mosquitoes are vectors of many pathogens, including West Nile virus, *Plasmodium* spp. (malaria) as well as filarial nematodes, which impose a huge burden on human and animal health. The development and multiplication of these pathogens within their biological vectors is highly temperature-dependent, hence temperature is one of the main drivers of transmission dynamics^2^. It is therefore important to understand the effect of temperature on both pathogen and vector as well as the interactions between the two.

Ectotherms such as mosquitoes can thermoregulate by selecting microhabitats^3^, and it has been shown that mosquitoes exhibit preferences for certain temperature ranges, also known as thermal preference^4–7^. Thermal preference is a thermoregulatory behaviour used to adapt to changing environmental conditions in order to increase survival. Furthermore, thermal preference might change upon infection, as shown for some insects. Locusts^8–10^ and house flies^11^ seek out warmer environments after infection to passively increase their body temperature (‘behavioural fever’). However, in other insects, the opposite trend was observed. For example, an infection of *Drosophila melanogaster* with *Pseudomonas aeruginosa* induced a preference for cooler temperatures (‘behavioural chill’), which in return increased survival of the flies^12^.

Very little is known about changes of thermal preferences in mosquitoes under the influence of an infection. A single such study investigated the effect of infections of *Anopheles stephensi* with fungal entomopathogens or with the rodent malaria parasite *Plasmodium yoelii*. No change in the preferred resting temperature was found for the mosquitoes that were tested on a gradient (14–38 °C) for multiple 30 min periods over five days after exposure to the pathogens^8^.

Here we extend the study of Blanford et al.^8^ by investigating the thermal behaviour of infected or immune-challenged mosquitoes, specifically the behaviour of *Aedes aegypti* exposed to Sindbis virus (SINV) and of *Ae. japonicus* exposed to the canine heartworm *Dirofilaria immitis*. In addition, mosquitoes were injected with Sephadex beads, triggering a melanisation immune response^13^. The mosquitoes’ thermal preferences were tested in the laboratory with a thermal gradient of 15–30 °C at different time points. The efficiency of melanisation in bead-injected mosquitoes was additionally quantified at various constant temperatures.

## Results

### Temperature preferences

#### *Aedes aegypti* exposed to SINV

In total, 741 female *Ae. aegypti* were exposed to blood spiked with SINV of which 417 (56%) died during the experimental time of 12 days. Of the surviving mosquitoes, 120 were used in the experiments. These were examined in 10 groups of 12 individuals (two groups per day) in a thermal gradient setup at different time points after the blood meal, and their position and the corresponding temperature after 15 min was noted. As determined by PCR, 62 mosquitoes (51.6%) were infected with SINV.

**Figure 1:**
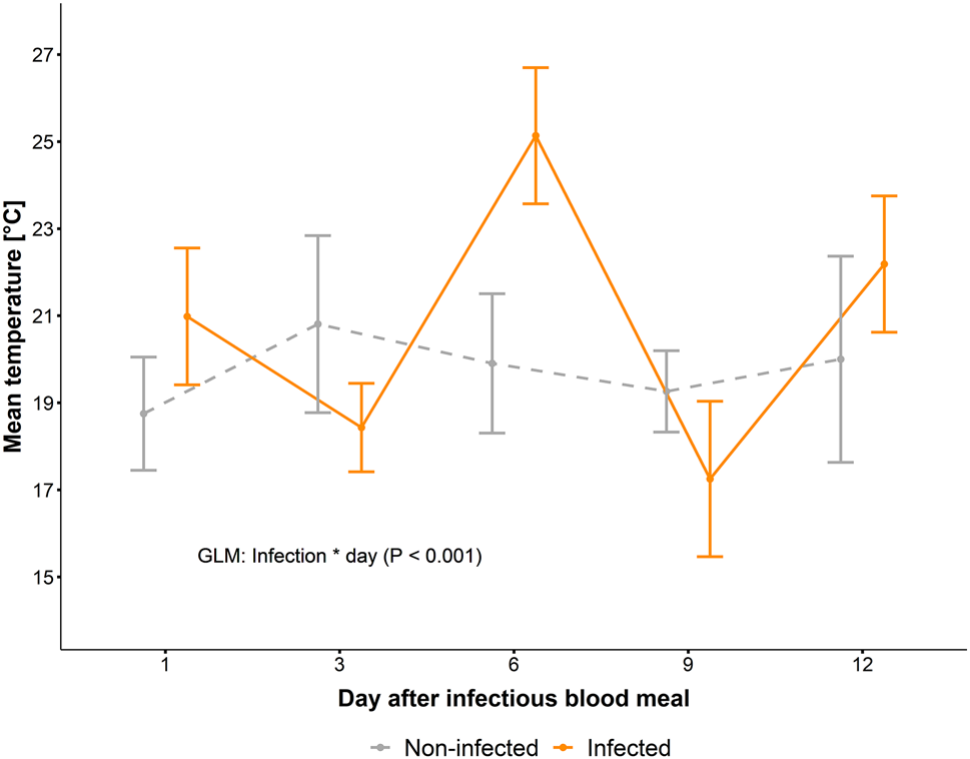
Preferred temperatures of *Aedes aegypti* infected with Sindbis virus (orange) vs. uninfected (grey at different days after the infectious blood meal. N_infected_ = 16, 11, 11, 4, 20; N_non-infeced_ = 8, 13, 13, 20, 4).

Non-infected *Ae. aegypti* mosquitoes preferred temperatures of 19.7 ± 5.1 °C, similar to the temperature preference of the infected mosquitoes when excluding day six (20.8 ± 5.1 °C). At day six, infected mosquitoes preferred a higher temperature of 25.1 ± 5.2 °C, which was 5.2 °C higher than the control mosquitoes on that day. This change in preferred temperature was detected by a significant interaction between temperature zone and experimental day (GLM, Chi^2^_4_ = 153.39, P < 0.001, Fig. 1). The preferred temperatures of infected mosquitoes differed significantly between treatments (infected and uninfected) and for the infected ones between day six and all other days (post-hoc tests: P < 0.001).

#### *Aedes japonicus* exposed to *Dirofilaria immitis*

Overall, 376 female *Ae. japonicus* were fed on infectious *D. immitis* blood of which 194 died (52%) during the 12 days of the experiment. Of the surviving mosquitoes, a total of 120 were used in the experiments as described above, of which 117 could be analysed. PCR analysis revealed an infection rate of 53.0% (62/117). Temperature preference was calculated for 62 infected and 55 non-infected mosquitoes.

**Figure 2:**
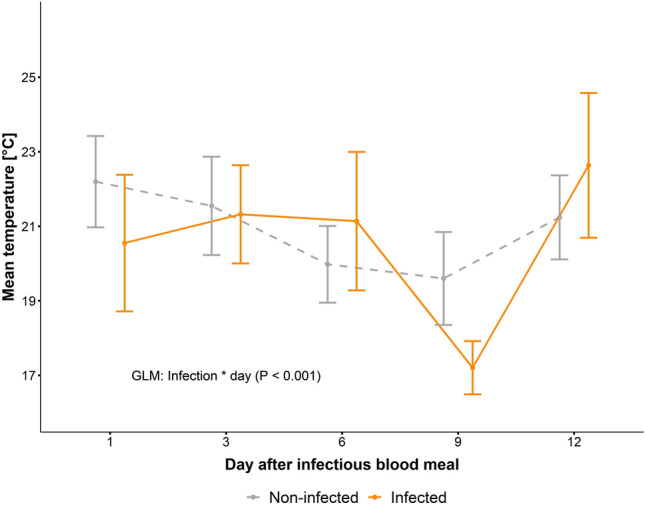
Preferred temperatures of *Aedes japonicus* infected with *Dirofilaria immitis* (orange) vs. uninfected (grey) at different days after the infectious blood meal. N_infected_ = 10, 14, 11, 16, 11; N_non-infeced_ = 12, 9, 13, 8, 13).

The overall preferred temperature of the non-infected mosquitoes was 20.9 ± 5.7 °C, similar to the temperature preference of the infected mosquitoes when excluding day nine (21.4 ± 5.8 °C). At day nine, however, infected mosquitoes preferred significantly cooler temperatures of 17.2 ± 2.9 °C (post-hoc, P < 0.001), as detected by a significant interaction between temperature zone and experimental day (GLM, Chi^2^_4_ = 85.94, P < 0.001, Fig. 2). This difference was significant between treatments at day nine (post-hoc, P < 0.001) as well as between days 6 & 9 and 9 & 12 (post-hoc, P < 0.001).

#### Bead-injected mosquitoes

Mosquitoes were either injected with Sephadex beads, injection solution (saline solution-injected control) or left untreated (non-injected control) and released in the gradient (15–30 °C), their position in each of the three sectors was determined four times within 24 h, and their survival was recorded (Fig. S1).

**Figure 3:**
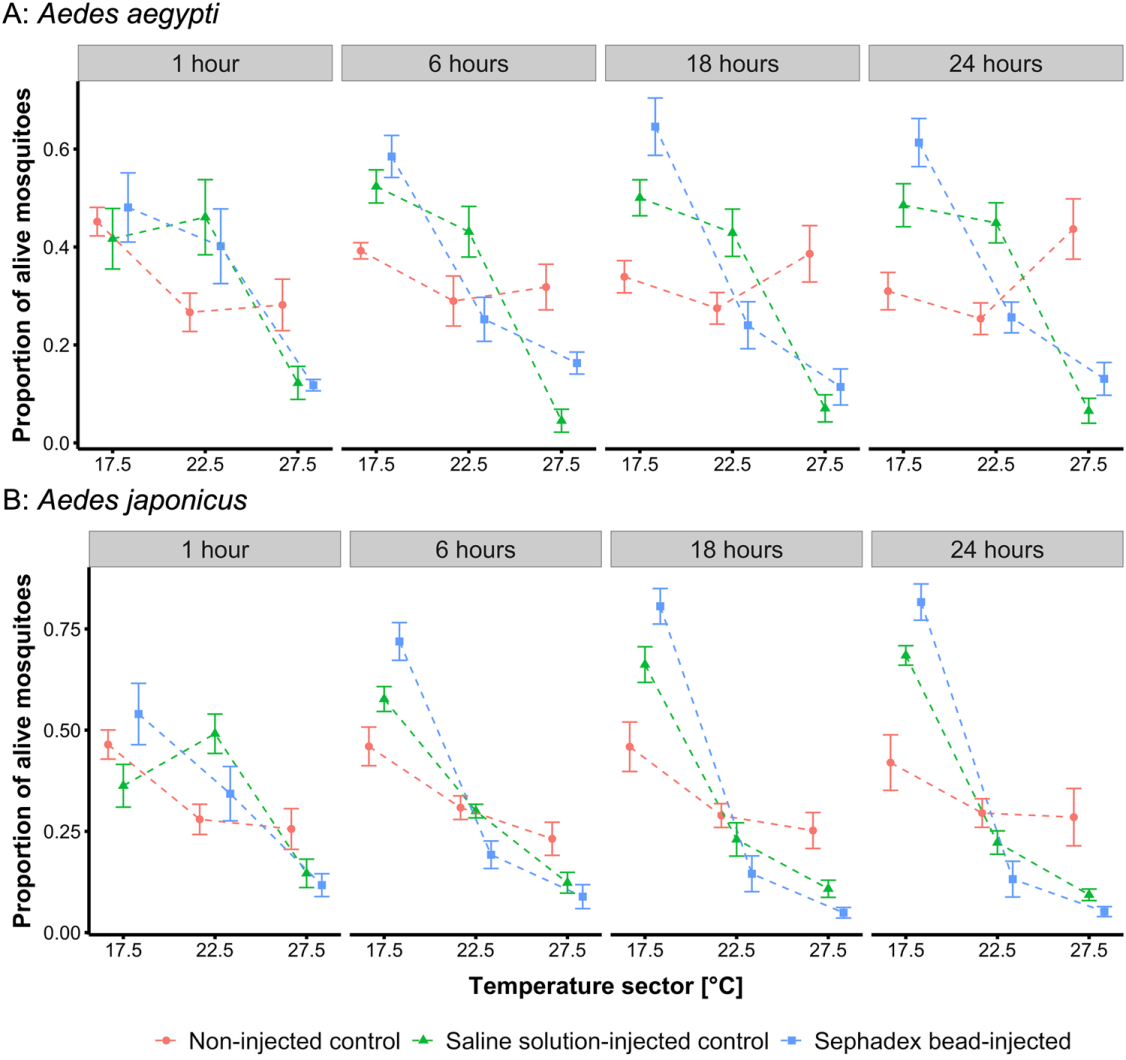
Preferred temperatures of *Aedes aegypti* (**A**) and *Ae. japonicus* (**B**) mosquitoes in a 15–30 °C gradient subdivided into three sectors, up to 24 h after release (non-injected control [red dots]), saline solution-injected [injected control, green triangles], and Sephadex bead-injected [blue rectangles]). Treatments were done max. 2 h before release. Given are means + /- standard errors over all replicates of the treatments. The total number of alive mosquitoes at each time point is given in Fig. S1.

Bead-injected *Ae. aegypti* showed a strong preference for the coolest sector (15–20 °C) over the duration of the experiments, and this trend became more pronounced over time (Fig. 3A). Overall, there were significant effects of the injection treatment (LMM, F_2_ = 3.40, P = 0.035) and the temperature sector (F_1_ = 79.53, P < 0.001) on the distribution of mosquitoes across the three sectors of the gradient (for statistical details see Table S1). After 24 h, 61% of the bead-injected mosquitoes rested in the coolest sector, while 26% were in the intermediate and only around 13% were found in the warmest sector (25–30 °C) (Fig. 3A). There was no significant difference between bead-injected and control-injected mosquitoes (e.g., at 24 h: LMM, t_48_ = -0.60, P = 0.554). The thermal preference of injected (both bead- and control-injected) and non-injected control *Ae. aegypti* differed except at time point 1 h. After 24 h, there was a significant difference between non-injected control and bead-injected (LMM, t_48_ = 5.79, P < 0.001) and control-injected mosquitoes (LMM, t_48_ = 5.19, P < 0.001).

The *Ae. japonicus* mosquitoes of all treatments showed an analogous behaviour in the thermal gradient as *Ae. aegypti* (Fig. 3B), with a more pronounced cold preference. Overall, there were significant effects of the injection treatment (LMM, F_2_ = 3.72, P = 0.026), the temperature sector (F_1_ = 83.23, P < 0.001), and hours in the gradient (F_1_ = 11.03, P = 0.001) on the distribution of mosquitoes across the three sectors of the gradient (for statistical details see Table S2). Thus, after 24 h, 82% of the bead-injected mosquitoes rested in the coolest sector, and 13% and 5% in the intermediate and warmest sector, respectively. There was no significant difference between bead-injected and control-injected mosquitoes (e.g., at 24 h: LMM, t_48_ = − 1.37, P = 0.178). The thermal preference of injected (both bead- and control-injected) and non-injected control *Ae. japonicus* differed except at time point 1 h. After 24 h, there was a significant difference between non-injected control and bead-injected (LMM, t_48_ = 4.97, P < 0.001) and control-injected mosquitoes (LMM, t_48_ = 3.61, P < 0.001).

### Melanisation response at constant temperatures

**Figure 4:**
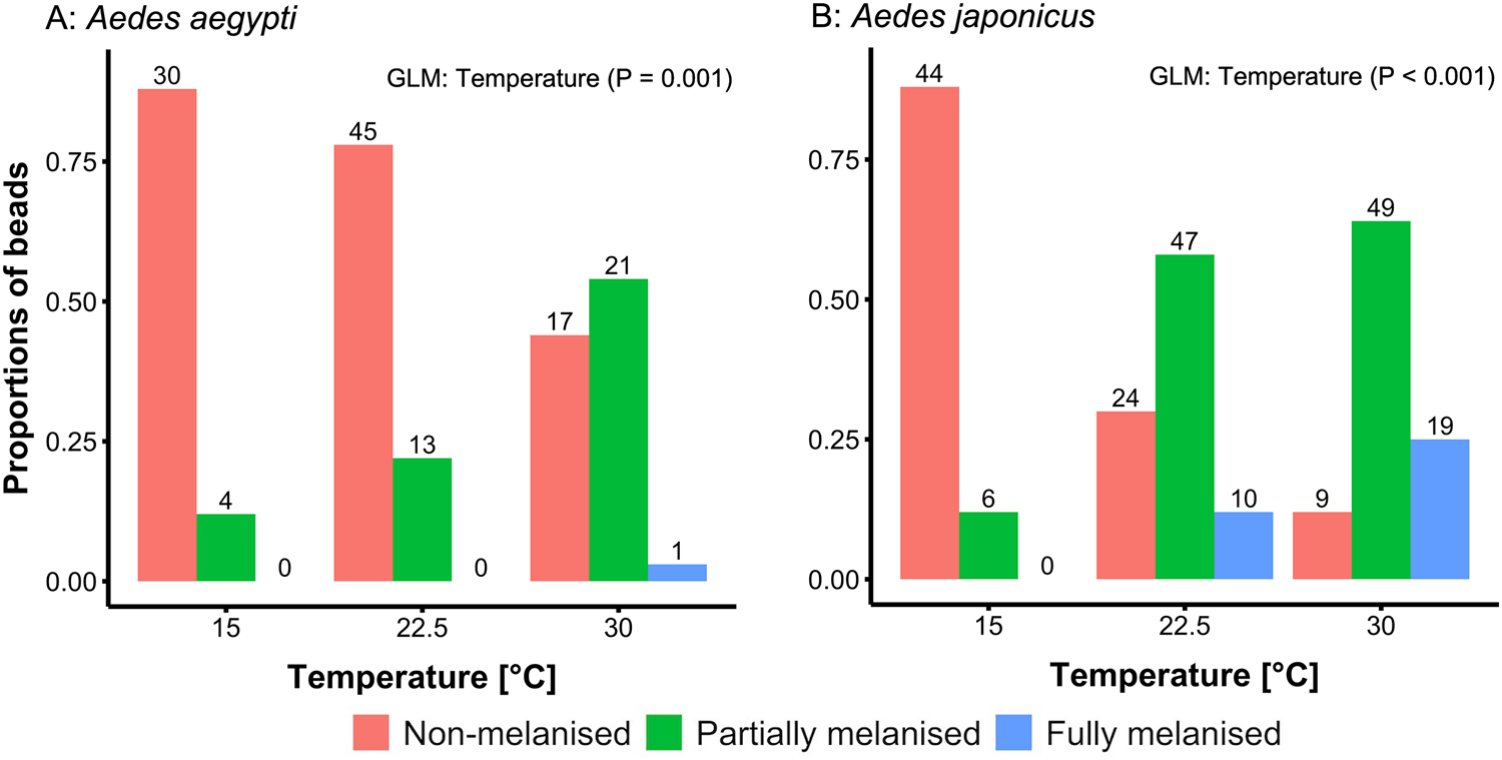
Proportions of non-melanised (red), partially melanised (green), and fully melanised (blue) Sephadex beads in female *Aedes aegypti* (**A**) and *Ae. japonicus* (**B**) after incubation at 15, 22.5 or 30 °C for 24 h. The total number of beads is indicated above the bars. For a more detailed illustration of the degrees of melanisation see Fig. S2.

In both species, temperature had a significant effect on bead melanisation (*Ae. aegypti*: GLM, Chi^2^_1_ = 10.25, P = 0.001; *Ae. japonicus*: GLM, Chi^2^_1_ = 40.91, P < 0.001), which was stronger at higher temperatures (Fig. 4). In *Ae. aegypti*, at 30 °C, 56% of the retrieved beads were partially or fully melanised and 44% were non-melanised, whereas at 15 °C, 88% of beads were not melanised, and there were no fully melanised ones (Fig. 4A). In *Ae. japonicus* a similar trend was observed, although the proportion of partially vs. fully melanised beads was higher at 22.5 °C (*Ae. japonicus*: 58% vs. 12%, *Ae. aegypti*: 22% vs. 0%) and at 30 °C (*Ae. japonicus*: 64% vs. 25%, *Ae. aegypti*: 54% vs. 3%) (Figs. 4B, S2) compared with *Ae. aegypti*. Survival of the treated mosquitoes was overall lower for *Ae. aegypti* (21%) than *Ae. japonicus* (34%) and was 100% in the control groups (Table S3).

## Discussion

Our experiments revealed that mosquitoes infected with SINV, *D. immitis*, or injected with Sephadex beads show a change in thermal preference. Both behavioural fever and chill were observed, depending on the treatment.

Following SINV infection, *Ae. aegypti* mosquitoes changed their temperature preference, favouring warmer temperatures on day 6 p.e. (Fig. 1). This behavioural fever has been demonstrated in other ectothermic species. For example, house flies infected with the fungal species *Beauveria bassiana* preferred temperatures approximately 3 °C higher than their uninfected counterparts^14^. Similar responses were observed in grasshoppers infected with microbial pathogens^15^, larvae of the moth *Galleria mellonella* exposed to *Aspergillus fumigatus*^16^, or honeybees exposed to a viral load^17^.

The preference for warmer temperatures observed in *Ae. aegypti* infected with SINV at day 6 p.e. might be due to the dissemination of the virus. In the study by Saredy et al.^18^, infection of midgut cells in *Ae. aegypti* and dissemination of SINV was investigated for the first 9 days p.e. by confocal analyses. Whilst midgut cells were infected as early as day 3 p.e., dissemination to legs was observed only after day 5 p.e. Correspondingly, RNAi, the innate immune response, was shown to be increased at day 7 p.e. as compared to days 1,2,3,4 p.e. when *Ae. aegypti* were kept at constant 28 °C^19^. Adelman et al.^20^ had shown that the RNAi response in *Ae. aegypti* was temperature dependent, i.e. was higher at 28 °C as compared to 18 °C. It could therefore be hypothesized that an increased body temperature, induced by a changed thermal preference, facilitates the immune response in mosquitoes at the time point of virus dissemination. The increase in preferred temperature was only observed at day 6 p.e., and it is unclear why it drops again after that. There is some evidence that dissemination continues until up to 14 days after infection^21^. To find out if the immune response to the disseminating virus decreases after dissemination starts, thus reversing the change in thermal preference, virus levels and RNAi immune response should be monitored in SINV-infected mosquitoes over longer time periods, e.g. 21 days.

In contrast, the temperature preference of *Ae. japonicus* mosquitoes shifted towards cooler temperatures (to ca. 17 °C from 21 °C; Fig. 2) when infected with *D. immitis,* but only at day 9 p.e. This could relate to melaninization, which is an immune response of mosquitoes against bacteria, protozoa, and filarial worms, including *D. immitis*^22–24^. A single study has addressed this aspect in *Ae. japonicus* exposed to *D. immitis*^25^. When examining dissected mosquitoes at days 1, 3, 4, 7, 10, 14, 21 p.e., melanised larvae (L1) were detected only after day 7 p.e., and therefore melanisation itself took place before day 7. As melanisation is a rapid process, i.e. within 24 h^13^, and as there was no shift in temperature preference at day 6 p.e., we conclude that the melanisation of L1 was probably not the causative agent for the observed temperature shift at day 9 p.e. Some studies suggest that melanisation is increased at cooler temperatures, which would be a possible explanation as to why mosquitoes infected with *D. immitis* prefer cooler temperatures^26–28^. However, all these studies were done with *Anopheles* mosquitoes, and in two of these studies, different temperature treatments than in our study were used. In one of these studies, the melanisation reaction in bead-injected mosquitoes using temperatures similar to the ones in the present study was investigated. Interestingly in this study, the proportion of partially melanised beads actually increased with temperature^27^. An alternative explanation for the shift in thermal preference at day 9 p.e. could be a stress response to the L3 larval stages, which were observed after day 7 in their developmental site (Malpighian tubules) and then move to the proboscis^25^.

Melanisation was investigated by injecting Sephadex beads into the thorax of *Ae. aegypti* and *Ae. japonicus*, revealing that melanisation increased with rising temperature (Fig. 4). This has been shown before by Murdock et al.^27^ in *An. stephensi*. Additionally, according to multiple studies, other factors of the immune response, such as haemocyte density, haemocyte count, nodules, and prophenoloxidase levels are higher at warmer temperatures, thus the insect’s immune response should be more efficient at higher temperatures^10,16,29,30^. Therefore, it would be beneficial for the insects to exhibit a behavioural fever (preference for warmer temperatures) to boost their immune system. Interestingly, in our study, the opposite reaction was observed with bead-injected mosquitoes preferring colder temperatures between 15 and 20 °C, when exposed to a temperature gradient from 15 to 30 °C, and this cold preference became stronger over time (between 1 and 24 h after inoculation; Fig. 3). The same was true for the injected control mosquitoes (only control solution injected). Non-injected control mosquitoes also displayed a cold preference, which was less pronounced and did not increase over time. In the case of *Ae. aegypti* this cold preference even diminished over time. As piercing the thorax causes a wound with a corresponding risk of desiccation, especially at elevated temperatures^31^, the cold preference of injected mosquitoes might be a strategy to escape the risk of desiccation. Melanisation is also involved in wound healing^32^ and is temperature-dependent^27^ suggesting that wounded mosquitoes should prefer warmer temperatures. The behaviour of injected mosquitoes on the thermal gradient observed in our study might therefore indicate a trade-off between the melanisation reaction and other factors affecting survival. Also, the cold preference of *D. immitis*-infected mosquitoes, as discussed above, might not be triggered by the melanisation response but rather by a stress response to the L3 larvae that are moving to the proboscis.

Finding both behavioural cold and warm preference in the same mosquito species (*Ae. aegypti*) suggests that this insect vector can adjust its thermal preferences according to the challenge encountered. Whereas a number of studies have described either ‘behavioural fever’ or ‘behavioural chill’ in ectotherms (see “Introduction”), we are not aware of another study addressing both behaviours in the same species upon different treatments.

Increasing body temperature alone does not seem to be sufficient to reduce pathogen loads in ectotherms below a certain level^14,15,17^. This raises the question whether behavioural fever is sufficient to boost the immune response or to kill the pathogens effectively.

Blanford et al.^8^ investigated the thermal behaviour of *An. stephensi* in a thermal gradient in response to infection with various pathogens. In contrast to the findings of our study, no changes in thermal preference were reported for mosquitoes infected with three different fungal entomopathogens and rodent malaria (*P. yoelii*). Rather, there was a strong preference for the release site in the middle of the setup. A possible reason might be the confined space of the experimental setup used (channels of 25 cm length, 2 cm width and 1 cm height), in which the mosquitoes were probably unable to fly in contrast to the setup used in the present study (60 × 30 × 4 cm). Further, Blanford et al.^8^ only studied the first five days of infection, whilst our study showed the first effects of infection with a virus on day 6 and with *D. immitis* on day 9. Therefore, future studies on thermal preferences of infected vectors should include the complete life cycle of the pathogen within a vector to uncover effects.

## Materials and methods

### Mosquitoes

A colony of *Ae. aegypti*, provided by the Institute Pasteur de Nouvelle Calédonie (IPNC), had been reared for over 30 generations in our laboratory. Adult mosquitoes had constant access to 10% sucrose solution and were fed three times a week with anticoagulated cow blood, obtained from a local slaughterhouse, with a Hemothek feeding system (Hemothek, Lancashire, UK). Eggs were collected by placing black oviposition cups (D = 7 cm, H = 6 cm; Luwasa, Allmendingen, Switzerland), lined inside with germination paper (Anchor Paper Co., Minnesota, USA), in the cages. The germination papers were flooded in buckets (5.4 L; Packstar, Nidau, Switzerland) with one litre of deionised water (dH_2_O), and the hatched larvae were fed with (finely ground) TetraMin fish food (TetraMin XL Flakes, Melle, Germany). Adult mosquitoes were transferred into 32 cm^3^ BugDorm insect cages (Taichung, Taiwan). *Aedes aegypti* of all stages were kept under constant 27 ± 1 °C, with a 16 h:8 h light:dark cycle (light on: 6:00 h, light off: 22:00 h) and 85% relative humidity (RH).

*Aedes japonicus* eggs were collected from several locations in central Switzerland by placing black oviposition cups lined with germination paper. Rearing to adults was done as described above. Adults were supplied with 5% glucose solution. All stages were kept under a constant temperature of 24 °C ± 1 °C, with a 16 h:8 h light:dark cycle (light on: 6:00 h, light off: 22:00 h) and 80% RH.

### Pathogens

SINV was supplied by Prof. Jan Lundström (Uppsala University, Sweden) in March 2014. The virus was passaged in Vero cells in Dulbecco’s modified eagle medium (DMEM) (Gibco No. 31885–023) and 10% foetal calf serum (BioConcept, Allschwil, Switzerland) at 37 °C and 5% CO_2_. Virus titer as determined by plaque assay was 1.48 × 10^7^.

*Dirofilaria immitis* microfilariae were provided prediluted at 3000 microfilariae / ml dog blood (mf/ml) by Elanco Animal Health, Basel, Switzerland. The *D. immitis* isolate was originally isolated in 2005 from an infected dog in Missouri (USA). Control blood stemmed from uninfected beagle dogs from the same supplier. The Li-heparin blood samples were stored at 4 °C until use.

### Thermal gradient

The thermal gradient used consisted of two thermal regulators (AHP-1200CPV, ThermoElectric Cooling America Corporation, Chicago, USA) connected by a conductive aluminium plate (91 × 30 × 2.5 cm^3^, TGB-5030, ThermoElectric) covered by a Plexiglas cover (60 × 30 × 4 cm^3^). The applied gradient ranged from 15 to 30 °C. Before the start of each experimental session, the gradient was established over at least 1 h. To keep RH constant, salt solutions were added to the far ends of the gradient as described before^4^. For analyses, the gradient was subdivided into five or three temperature zones, each 12 cm or 20 cm wide, respectively. Temperature was measured with three dataloggers (MSR145, MSR, Seuzach, Switzerland), one located in the middle and one at each of the two extreme ends.

### Exposure to SINV or *D. immitis*

Around 800 female mosquitoes aged five to twelve days were starved for 24 h and then allowed to take a blood meal by forced feeding^33^ on a Hemotek with a Parafilm membrane. The blood meals contained either SINV with a titer of 1.48 × 10^6^ in bovine blood (i.e., 1:10 dilution from stock) or 3000 mf/ml of *D. immitis* in dog blood. Mosquitoes were allowed to feed for 2 h. Every 15 min, a pulse of CO_2_ was provided by gently breathing over the feeder. After feeding, fully engorged mosquitoes were kept in BugDorms at rearing conditions over night. Gloves were worn throughout the procedure. Forceps, Hemoteks and the force feeders were cleaned with Virkon S (Arovet AG, Dietikon, Switzerland) and 70% ethanol. Garbage in contact with blood and all gloves were autoclaved before disposal.

The temperature preference of the mosquitoes was assessed at days 1, 3, 6, 9, and 12 post feeding. On each day in the afternoon (between 13:00 h and 16:00 h), two tests were run with twelve mosquitoes each. Treatment groups and orientation of the temperature gradient alternated between the experimental days. Mosquitoes were released in the middle of the gradient. After 15 min, the behavioural setup was flooded with CO_2_ until all mosquitoes were motionless for at least 10 s. The location of mosquitoes on the gradient was recorded and mosquitoes individually stored in Eppendorf tubes at − 80 °C for SINV- and − 20 °C for *D. immitis-*exposed ones. After each run, the cover of the setup was lifted for one minute to let the CO_2_ escape. Thereafter, the gradient was re-established over 15 min for the next run.

### Injection with Sephadex beads

Injections followed the method described by Barreaux et al.^13^, with small changes. Microcapillary glass tubes were heat-pulled using a needle-puller (Model: cp-10, Narishige group, London, United Kingdom). The tips were broken off under a binocular microscope to obtain a sharp and very small opening. A small amount (~ 9 mg) of autoclaved, negatively charged Sephadex CM C-25 beads (diameter 40–120 μm; Sigma-Aldrich, Steinheim, Germany) were added to a petri dish and stained with 5 ml injection solution (1.3 mM NaCl, 0.5 mM KCl, 0.2 mM CaCl_2_ [pH 6.8], 0.001% methyl green).

Female mosquitoes were anesthetized in groups of 5–10 individuals by transferring them into a 50 ml Falcon tube by mouth aspirator and placing them on ice for 1–5 min. Then, one mosquito at a time was placed on a filter paper under the microscope with its left side facing upwards. With a pipette bulb and making use of the capillary effect, one stained, small (diameter 40–60 µm) bead with approx. 0.1 µl injection solution was aspirated into the glass capillary. The needle was then manually pierced through the left side of the anesthetized mosquito’s thoracic cavity cuticle, and the bead was injected^13^.

### Experimental procedure for bead-injected mosquitoes in the thermal gradient

For each treatment (Sephadex bead-injected, injection solution-injected control, non-injected control) six replicates were completed, resulting in 18 trials. For each trial, approximately 50 mosquitoes (2–5 days old) were selected around 10:00 h. After the treatment, the mosquitoes were placed in a plastic cup with direct access to a sugar solution-soaked cotton ball until the experiment in the behavioural gradient started at 13:00 h. The order of the three treatments was randomized, and the direction of the gradient was reversed for half of the replicates of each treatment. The mosquitoes were provided with cotton balls soaked with sugar solution in each of the three sectors of the thermal gradient. The number of dead and live mosquitoes in each of the three sectors were counted after 1, 6, 18 and 24 h (± 1 h). After the last count, all mosquitoes were killed with CO_2_.

### Melanisation of beads at constant temperatures

Mosquitoes (bead-injected, non-injected controls) were incubated for 24 h at 15, 22.5 or 30 °C, respectively. For each of the three temperature treatments, several replicates (*Ae. aegypti*: five at 15 and 30 °C, four at 22.5 °C, *Ae. japonicus*: five at 15, 22,5 and 30 °C) were completed, with ca. 50 bead-injected and 10–25 non-injected control individuals at a time. After injection, the mosquitoes were transferred into one of two 17 cm^3^ BugDorm insect cages according to their treatments around 12:00 h. The mosquitoes had constant access to cotton balls soaked with sugar (*Ae. aegypti*: 10% sucrose; *Ae. japonicus*: 5% glucose). Wet paper towels and trays with water ensured that the relative humidity in the incubator was constantly above 60%.

After 24 h, dead and live mosquitoes were counted. The surviving bead-injected mosquitoes were collected and frozen at − 20 °C until analyses. Beads were retrieved after careful dissection from the thorax of the insects in a Petri dish, containing dissection solution (1.3 mM NaCl, 0.5 mM KCl, 0.2 mM CaCl_2_ [pH 6.8], 0.01% methyl green). The degree of melanisation was visually estimated under a binocular at percentage intervals (0%, 1–20%, 21–49%, 50%, 51–80%, 81–99%, 100%). For the final statistical analyses, three categories were considered: non-melanised, partially melanised, and fully melanised (Fig. S3).

### PCR

Stored mosquitoes were dissected by removing the abdomen from the rest of the body using a pair of sterile stainless-steel forceps and putting both parts into separate Eppendorf tubes. These tubes were then stored at − 80 °C for SINV- and − 20 °C for *D. immitis-*exposed mosquitoes.

RNA extraction was accomplished with the QIAamp Viral RNA Mini kit (Qiagen, Hilden Germany), DNA extraction with the QIAamp DNA Mini kit (Qiagen). Both extractions were performed according to the manufacturer’s instructions (viral RNA protocol & DNA extraction from insect protocol; both from the manufacturer Qiagen). RNA samples were stored at − 80 °C and DNA samples at − 20 °C until PCR.

Amplification of SINV RNA and *D. immitis* DNA was performed by real-time PCR (qPCR) in a CFX96 Touch Real Time System (Bio-Rad Laboratories, Cressier, Switzerland). On each well plate, one experimental day was analysed together with one positive control (infected blood) and two negative controls (dH_2_O and uninfected blood).

SINV RNA was detected by using the iTaq Universal Probes One-Step Kit (Bio-Rad Laboratories, Switzerland). Each well contained 5 µl of extracted RNA or dH_2_O, 12.5 µl one-step reaction mix, 4 µl RNAse free water, 1 µl of each primer CBSINVF (7.5 µM) 5′-CTGY TCA TAC GGG AAC ATT CC-3′ and CBSINVR (7.5 µM) 5′-CAC TGA CWTC ACA TTT GAC TGT TGA-3′, 1 µl of probe CBSINVP (5 µM) FAM—5′-CCG AAC GCT GCC TTT ATC AGG ACA TC-3′—BHQ-1, and 0.5 µl iScript reverse transcriptase. The primers and the probe were designed and provided by Laboratory Spiez, Switzerland. The cycler was set to 50 °C for 10 min, 95 °C for 5 min followed by 50 cycles of 95 °C for 15 s and 56 °C for 30 s.

qPCR for detection of *D. immitis* DNA was performed with an iCycler (Bio-Rad Laboratories, Cressier, Switzerland) as previously described^25^. Each well contained 5 µl of extracted DNA or dH_2_O, 10 µl iQ Supermix (Bio-Rad Laboratories), 2 µl dH_2_O, 1 µl Diro-f (7.5 µM) 5′-GGT GTT TGG GAT TGT TAG TGA A-3′, 1 µl Diro-r (7.5 µM) 5′-CAG CAA TCC AAA TAG AAG CAA-3’, and 1 µl Diro-p (5 µM) 5′-FAM-TCT GGC CAA ACA AAC GAT CCT TAT CA-TAMRA-3′. Reaction conditions were 95 °C for 2 min and then 40 cycles of repeating 15 s at 95 °C followed by one minute at 55 °C.

### Data analysis

For data analysis of the infection experiments, mean preferred temperature was inferred assuming a linear temperature distribution across the gradient. To model the infections, a generalised linear model (GLM) with negative binomial family was used with day, state of infection, orientation of gradient, and the interaction between the first two, as explanatory variables. For all significant factorial variables and all significant interactions, Tukey post-hoc tests were performed.

For the data analysis of the thermal preference experiments with injected mosquitoes, the proportion of live mosquitoes was counted in every sector at every time point. For both *Ae. aegypti* and *Ae. japonicus*, separate linear mixed effects models (LMM) were performed. The injection treatment (bead, solution, control), sector of the gradient, hours in the gradient and their interactions were used as explanatory variables, and the trial was added as a random effect to account for repeated counts within each trial.

To analyse the melanisation of beads at different constant temperatures, a GLM with negative binomial distribution was performed. Temperature and day of the experiment were added as explanatory variables.

Analysis of all data was performed in R v. 4.1.0^34^. The lme4 package was used for modelling v. 1.1-31^35^, the ggplot2 package for visualisation v.3.4.0^36^ and the dplyr package for data clean up v.2.2.1^37^.

### Supplementary Information

Supplementary Information.

## Data Availability

Code and data can be found under: 10.5281/zenodo.10417714.
