# Supplementary material for: Altered thermal preferences of infected or immune-challenged Aedes aegypti and Aedes japonicus mosquitoes
Source: Sci Rep. 2024 Jun 5;14:12959. doi: 10.1038/s41598-024-63625-4 (PMC11153553; doi:10.1038/s41598-024-63625-4)
Supplement: Supplementary file 1 — Supplementary Information. [file 41598_2024_63625_MOESM1_ESM.pdf]

Supporting information

Altered thermal preferences of infected and immune-challenged *Aedes aegypti* and *Aedes japonicus* mosquitoes

David O. H. Hug[#], Raphaela Gretener-Ziegler[#], Raffael I. Stegmayer, Alexander Mathis, Niels O. Verhulst[*]

National Centre for Vector Entomology, Institute of Parasitology, Vetsuisse and Medical Faculty, University of Zürich, Zürich, Switzerland

[#]These authors contributed equally

*Corresponding author

National Centre for Vector Entomology, Institute of Parasitology, Vetsuisse and Medical Faculty, University of Zürich, Winterthurerstr. 266A, 8057 Zürich, Switzerland

E-mail address: niels.verhulst@uzh.ch (N.O. Verhulst)

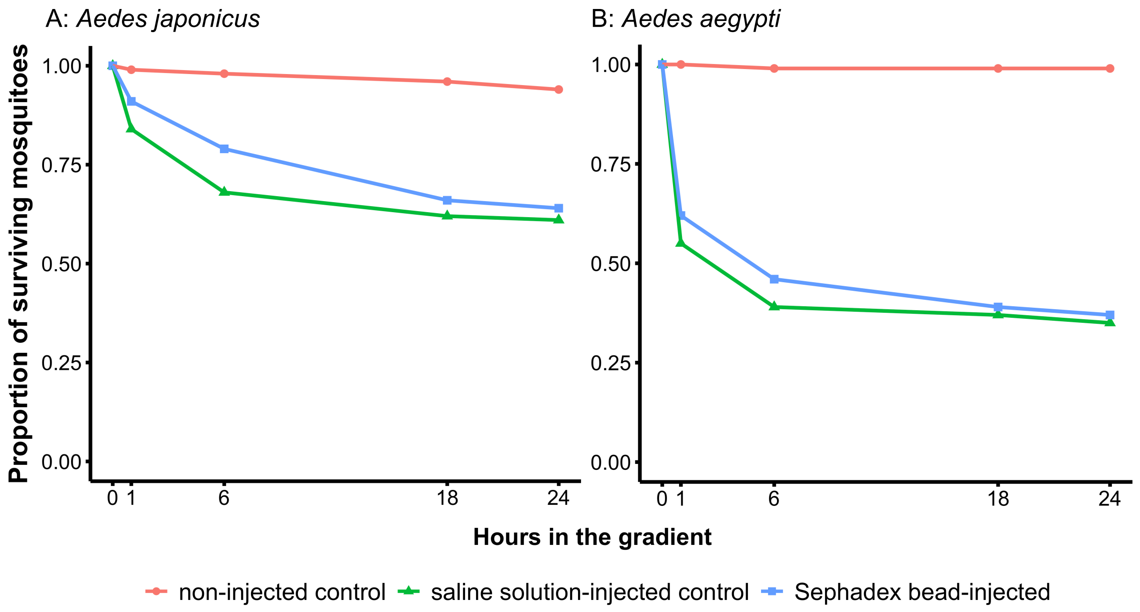

Figure S1: Survival curves of *Aedes japonicus* (A) and *Ae. aegypti* (B) in the injection experiment over time in the thermal gradient (15 – 30 °C). Mosquitoes were either non-injected (control, red dots), saline solution-injected (control, green triangles), or bead-injected (treatment, blue rectangles). The following numbers of mosquitoes were released: *Ae. japonicus*: N (non-injected control) = 279, N (saline solution-injected control) = 309, N (Sephadex bead-injected) = 317; *Ae. aegypti*: N (non-injected control) = 286, N (saline solution-injected control) = 279, N (Sephadex bead-injected) = 304.

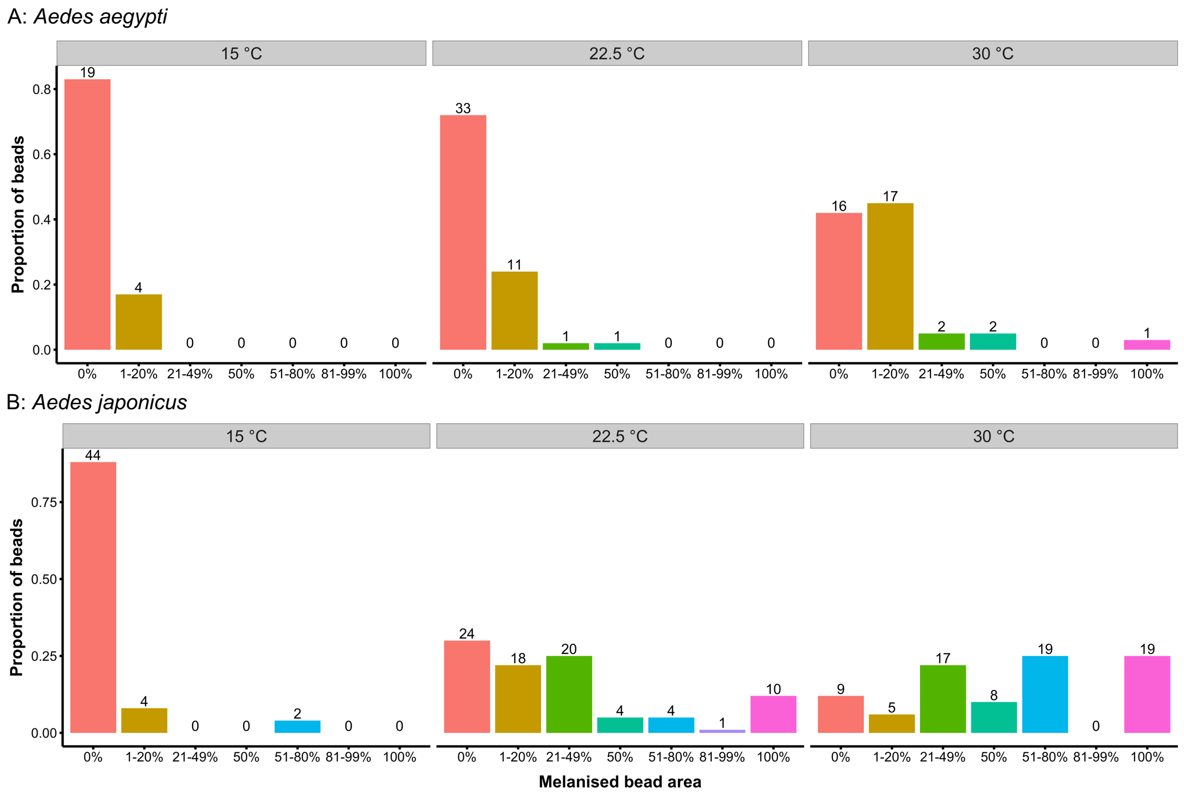

Figure S2: Estimated percentage of bead area melanised in *Aedes aegypti* (A) and *Ae. japonicus* (B) after incubation at 15, 22.5 or 30 °C for 24 h. One bead was injected per mosquito, and the total number of beads in each category is indicated above the bars. For analyses, three categories were determined (0: non-melanised; 1: partially melanised; 2: fully melanised; see Figure 4, Figure S3).

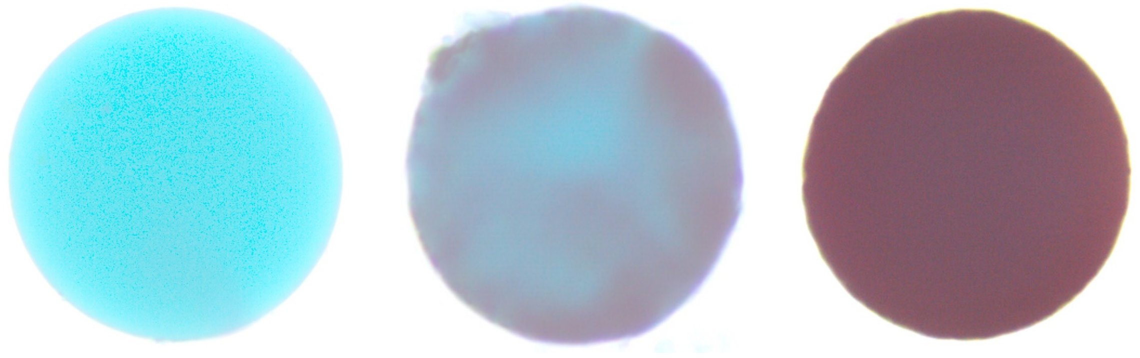

Figure S3: Examples of Sephadex beads with different melanisation intensities: non-melanised, partially melanised and fully melanised. The pictures were taken with a light microscope (Leica DM1000 LED, Leica Microsystems) and a camera (Leica DFC400, Leica Microsystems) at 400x magnification.

**Variables**	**Degrees of freedom**	**F-value**	**P-value**
Treatment	2	3.40	**0.035**
Sector	1	79.53	**< 0.001**
Hours	1	0.18	0.673
Treatment*Sector	2	3.96	**0.020**
Treatment*Hours	2	5.61	**0.004**
Sector*Hours	1	0.21	0.648
Treatment*Sector*Hours	2	6.54	**0.002**

Table S1: Results of the linear mixed effects model (LMM) on the thermal preference of Sephadex bead-injected, injection solution-injected and non-injected *Ae. aegypti*. The proportion of alive mosquitoes was counted in every sector of the 15-30 °C gradient after 1, 6, 18 and 24 hours. The injection treatment (Sephadex bead, injection solution, non-injected control), sector of the gradient, hours in the gradient and their interactions were used as explanatory variables, and the trial was added as a random effect to account for repeated counts within each trial.

**Variables**	**Degrees of freedom**	**F-value**	**P-value**
Treatment	2	3.72	**0.026**
Sector	1	83.23	**< 0.001**
Hours	1	11.03	**0.001**
Treatment*Sector	2	4.34	**0.014**
Treatment*Hours	2	5.05	**0.007**
Sector*Hours	1	12.87	**< 0.001**
Treatment*Sector*Hours	2	5.90	**0.003**

Table S2: Results of the linear mixed effects model (LMM) on the thermal preference of Sephadex bead-injected, injection solution-injected and non-injected *Ae. japonicus*. The proportion of alive mosquitoes was counted in every sector of the 15-30 °C gradient after 1, 6, 18 and 24 hours. The injection treatment (Sephadex bead, injection solution, non-injected control), sector of the gradient, hours in the gradient and their interactions were used as explanatory variables, and the trial was added as a random effect to account for repeated counts within each trial.

**Temperature**	***Aedes aegypti***		***Aedes japonicus***	
**(°C)**	**bead-injected**	**control**	**bead-injected**	**control**
15	38 (15%)	55 (100%)	61 (23%)	124 (100%)
22.5	71 (30%)	40 (100%)	94 (41%)	120 (100%)
30	51 (19%)	60 (100%)	89 (39%)	127 (100%)

Table S3: Survival of mosquitoes in the injection experiments at the different constant temperatures (15, 22.5 or 30 °C). Given are the absolute numbers of surviving mosquitoes after 24 h as well as survival rates as a percentage for Sephadex bead-injected vs. non-injected mosquitoes.
